# A Rare Central Venous Catheter Malposition: A Case Report

**DOI:** 10.5812/aapm.16049

**Published:** 2014-02-05

**Authors:** Ali Asghar Moeinipour, Ahmad Amouzeshi, Marjan Joudi, Mehdi Fathi, Saeed Jahanbakhsh, Saeed Hafez, Azra Izanloo, Mahmood Khorsand

**Affiliations:** 1 Department of Cardiac Surgery, Imam reza Hospital, Faculty of Medicine, Mashhad University of Medical Sciences, Mashhad, Iran; 2 Department of Cardiac Surgery, Imam reza Hospital, Faculty of Nursing, Mashhad University of Medical Sciences, Mashhad, Iran; 3 Department of Radiologic Technology, Faculty of Paramedicine Sciences, Mashhad University of Medical Sciences, Mashhad, Iran

**Keywords:** Central Venous Catheter, Venous Pressure, Catheters, Vascular Access Devices, Catheters, Indwelling

## Abstract

**Introduction:**

Central venous catheter placement is a routine procedure for the management of critically ill patients; however, it is important to ensure its proper placement. A central venous catheter malposition may cause various complications, some of which can be fatal.

**Case Presentation:**

We report an unexpected malposition of a catheter in the left internal jugular vein, where it entered into the left internal mammary vein.

**Conclusions:**

We think one of the influential factors for leading a guidewire and catheter into a nominated vein may be the left sided bevel of the needle at the time of internal jugular vein needle and catheter insertion. We were required to continue going towards the subclavian vein and accidentally turned into the left internal mammary vein.

## 1. Introduction

Central venous catheterization is a simple, relatively inexpensive method of assessing a patient's circulating blood volume, cardiac status and vasomotor tone, moreover, it is an essential component of modern-day critical care (1). Central venous catheterization plays many important roles in anesthesia and ICU care, including the measurement of right atrium pressure. In addition, it is used for any cardiac patient requiring a cardiopulmonary bypass circuit or any condition in which large amounts of volume or blood loss are expected. On the other hand, a central venous catheter is also used for blood product infusion and vasopressor drug administration. In extraordinary situations, it may be used for further pulmonary artery catheter placement, implantation of an atrial pacemaker, or right heart mechanical assist devices. Although it is a relatively safe procedure, multiple complications have been described both during its placement and in the maintenance of the catheter. Sometimes malposition may cause serious problems, even fatal events. Malposition of the catheter tip is one of them. This report concerns a rare malposition of a central venous catheter tip in the left internal mammary vein.

## 2. Case Presentation

**Figure 1.**
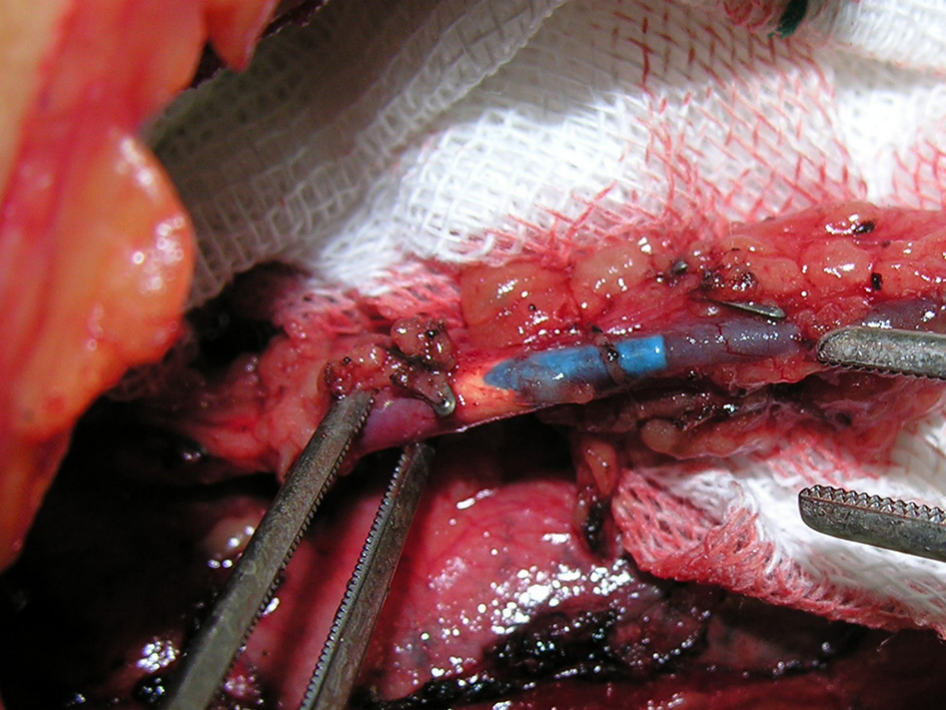
Catheter in the Left Internal Mammary Vein

The patient was a 54-year-old man, who was a candidate for coronary artery bypass grafting. He had a history of cardiac arrest, following acute myocardial infarction, on the day before his surgery. After cardiopulmonary resuscitation, an urgent angiography was done and this showed that the left main and left circumflex arteries had significant stenosis. Therefore, the patient was a candidate for coronary bypass graft surgery. Induction of anesthesia and insertion of central venous cannulation were carried out by an expert anesthesiology team. For preloading and induction of the anesthesia, the right external jugular vein was used as the peripheral venous access, because we were unable to find any peripheral access on the limbs due to the patient’s morbid obesity. We then selected the left internal jugular vein for central venous cannulation. Cannulation was conducted without difficulty, and after blood aspiration a catheter was fixed at 15 cm. We continued normal saline infusion through the central cannulated vein and this procedure went smoothly. We did not use central venous waveforms to measure central venous pressure or catheter position confirmation as usual. After a sternotomy, the surgeon started to release the left internal mammary artery to use for grafting. Surprisingly, he found that the catheter had entered into the left internal mammary vein (Figure 1). 

We extracted the catheter in order to prevent venous occlusion and continued fluid and drug replacement from the right external jugular vein. We placed a central venous catheter through the internal jugular vein after the surgery had been completed.

## 3. Conclusions

Central venous catheterization is important because it helps measure central venous pressure (CVP), for the purpose of adequate replacement of blood and fluid loss, during the preoperative period (2). Therefore, central venous cannulation (CVC) in heart surgery is used to enable direct venous access for the infusion of intravascular volumes and fluids, in tropes and measurement of the right atrial pressure. The internal jugular vein is the most common site which anesthesiologists use for this purpose, and its selection provides the greatest security. The most accurate location of the tip of the catheter for measuring central venous pressure is at the junction of the superior vena cava to the right atrium. More than 15% of patients with vein catheter insertion will experience catheter complications. Mechanical complications occur in 5-19% of patients, infectious complications in 5-26 % and thromboembolic complications in 2-26% of patients (3). Malposition of the catheter may induce complications that can potentially be fatal. The incidence of catheter malposition has been reported to be 3 to 4%.The most common reports of malposition refer to subclavian vein cannulation (4), which is catheterization entrance into the internal jugular vein (IJV) (2).

Turi reported a case of catheter placement at the anterior mediastinum, following superior vena cava perforation (5). Trunjillo described two cases of accidental placement of a central venous catheter in the left pericardiophrenic vein (6). Leang reported a case of accidental placement of the central venous catheter in the plural cavity (7). Gentili described three cases, one of which had malposition in catheterization. They reported an malposition of a catheter left internal thoracic vein (8). Tomar reported a case of internal rotation inside the vessel which was an example of malposition of a central venous catheter (2).

Some other malpositions involved the turning of a catheter into the right internal jugular vein following right subclavian vein cannulation (9) or entrance into the left upper intercostal vein. In some cases malposition may be expected due to anatomical anomalies such as the presence of a left superior vena cava (1). Our patient had no anatomical abnormalities and the presented malposition could have been entirely incidentally. 

We have reported a very rare malposition of the central venous cannula. The internal jugular vein joins the innominate vein and then, the innominate vein joins the subclavian vein, which the left internal mammary vein is one of its divisions. Taking into account the previously mentioned anatomy, we think that one of the influential factors for leading the guidewire and catheter to the innominate vein, may be the left sided bevel of the needle at the time of internal jugular vein needling and catheterization which obligates continuation towards the subclavian vein and that is when the catheter can accidentally be turned into the left internal mammary vein.

Although insertion of the catheter in this case was carried out by an expert cardiac anesthesiologist, it seems that observing central venous waveforms may be a suggestive guide for the correct positioning of the catheter, which we did not use in this case. On the basis of recent studies, ultrasonography is used to detect the internal jugular vein and reduce the complications of catheterization (1, 10). Therefore, it would be better to use ultrasound in a catheterization procedure. Furthermore, some cardiac surgeons prefer not to insert a central venous catheter through the left internal jugular or left subclavian vein, due to the likelihood of accidental left mammary artery puncture. In conclusion, we suggest that our colleagues choose the right internal jugular vein as the first option for this procedure.
